# Living joint prosthesis with in-situ tissue engineering for real-time and long-term osteoarticular reconstruction

**DOI:** 10.1016/j.bioactmat.2025.01.036

**Published:** 2025-02-26

**Authors:** Wei Sun, Hongwei Wu, Yiyang Yan, Xianzhu Zhang, Xudong Yao, Rui Li, Jingyi Zuo, Wenyue Li, Hongwei Ouyang

**Affiliations:** a Department of Sports Medicine of the Second Affiliated Hospital, and Liangzhu Laboratory, Zhejiang University School of Medicine, Hangzhou, China; b Dr. Li Dak Sum & Yip Yio Chin Center for Stem Cells and Regenerative Medicine, Zhejiang University School of Medicine, Hangzhou, China; c Zhejiang University-University of Edinburgh Institute, Zhejiang University School of Medicine, Haining, China; d China Orthopedic Regenerative Medicine Group (CORMed), Hangzhou, China; e Department of Orthopedics, The First Affiliated Hospital, Zhejiang University School of Medicine, 79 Qingchun Rd, Hangzhou, 310003, China; f Center of Regenerative and Aging Medicine, the Fourth Affiliated Hospital of School of Medicine, and International School of Medicine, International Institutes of Medicine, Zhejiang University, Yiwu, 322000, China; g Department of Orthopedics, The Fourth Affiliated Hospital of School of Medicine, and International School of Medicine, International Institutes of Medicine, Zhejiang University, Yiwu, 322000, China

**Keywords:** Large osteoarticular defect, Living prosthesis, Biological reconstruction

## Abstract

The reconstruction of large osteoarticular defects caused by tumor resection or severe trauma remains a clinical challenge. Current metal prostheses exhibit a lack of osteo-chondrogenic functionality and demonstrate poor integration with host tissues. This often results in complications such as abnormal bone absorption and prosthetic loosening, which may necessitate secondary revisions. Here, we propose a paradigm-shifting “living prosthesis” strategy that combines a customized 3D-printed hollow titanium humeral prosthesis with engineered bone marrow condensations presenting bone morphogenetic protein-2 (BMP-2) and transforming growth factor–β3 (TGF-β3) from encapsulated silk fibroin hydrogels. This innovative approach promotes *in situ* endochondral defect regeneration of the entire humeral head while simultaneously providing immediate mechanical support. In a rabbit model of total humerus resection, the designed “living prosthesis” achieved weight, macroscopic and microscopic morphologies that were comparable to those of undamaged native joints at 2 months post-implantation, with organized osteochondral tissues were regenerated both around and within the prosthesis. Notably, the “living prosthesis” displayed significantly higher osteo-integration than the blank metal prosthesis did, as evidenced by a 3-fold increase in bone ingrowth and a 2-fold increase in mechanical pull-out strength. Furthermore, the "living prosthesis" restored joint cartilage function, with rabbits exhibiting normal gait and weight-bearing capacity. The successful regeneration of fully functional humeral head tissue from a single implanted prosthesis represents technical advance in designing bioactive bone prosthesis, with promising implications for treating extreme-large osteochondral defects.

## Highlights

• One-time surgery with a customized “living prosthesis"’ for lifelong use.
• Bioactive metal prosthesis-guided in situ tissue regeneration with sufficient mechanical support.
• Hierarchical regeneration of cartilage and osteochondral tissue of entire humeral head joints.

## Graphical abstract

**Figure:**
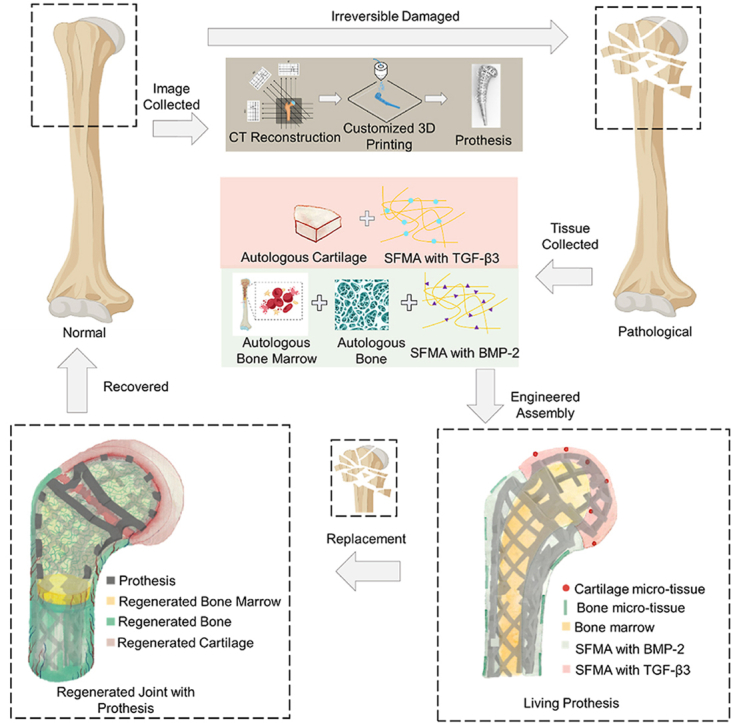

## 1 Introduction

The clinical management of osteoarticular defects arising from aging, trauma, infection, tumors, and deformities poses significant challenges, particularly in cases involving large-scale defects such as late-stage osteoarthritis [[1], [2], [3], [4], [5]]. Total joint replacement (TJR) has emerged as the most viable treatment option for extensive osteoarticular defects, particularly in extremely severe cases [6,7]. However, the majority of current prostheses remain bioinert, relying primarily on mechanical anchors for integration into the host tissue. This approach often leads to a range of complications, including loosening and fracture, which lead to a limited lifetime of 20 years[[8], [9], [10]]. With the prolongation of the average human lifespan, most TJR patients may necessitate complex secondary surgical interventions.

Recently, a tissue engineering strategy that combines scaffolds and growth factors has demonstrated significant regenerative potential for smaller defects[[11], [12], [13], [14], [15]]. However, their relatively low stiffness hinders their application in repairing large-scale weight-bearing joints. Given these limitations, there is an urgent need for prostheses that offer immediate mechanical support while possessing regenerative capacity in the treatment of large osteoarticular defects. The development of such prostheses represents a significant advancement in addressing the challenges associated with traditional bioinert prostheses and has the potential to transform the field of orthopedic surgery.

Titanium (Ti) has long been the material of choice for orthopedic implants due to its excellent mechanical properties, corrosion resistance, and osseointegration capabilities. Its biocompatibility and the ability to form a stable bond with bone tissue make Ti a cornerstone in implant design [16,17]. However, the long-term usage of Ti implants could be further enhanced by incorporating with the surrounding tissue [18]. To achieve this goal, bioactive coatings that promote cell adhesion, differentiation, and tissue formation are required. As joints are under repeated movements, 10.13039/100028664SFMA hydrogels serves as an ideal candidate for providing tunable mechanical properties, good biocompatibility, and the capacity to support cell growth and differentiation, particularly in applications requiring flexibility and resilience [[19], [20], [21]]. BMP-2 is a well-known osteogenic growth factor that promotes bone formation, while TGF-β3 regulates chondrogenesis and extracellular matrix synthesis [22,23]. By incorporating these elements, we aimed to construct a prosthesis with instant mechanical strength while providing guidance for the correct regeneration of cartilage and bone tissues in the corresponding parts of the joints.

**Scheme 1:**
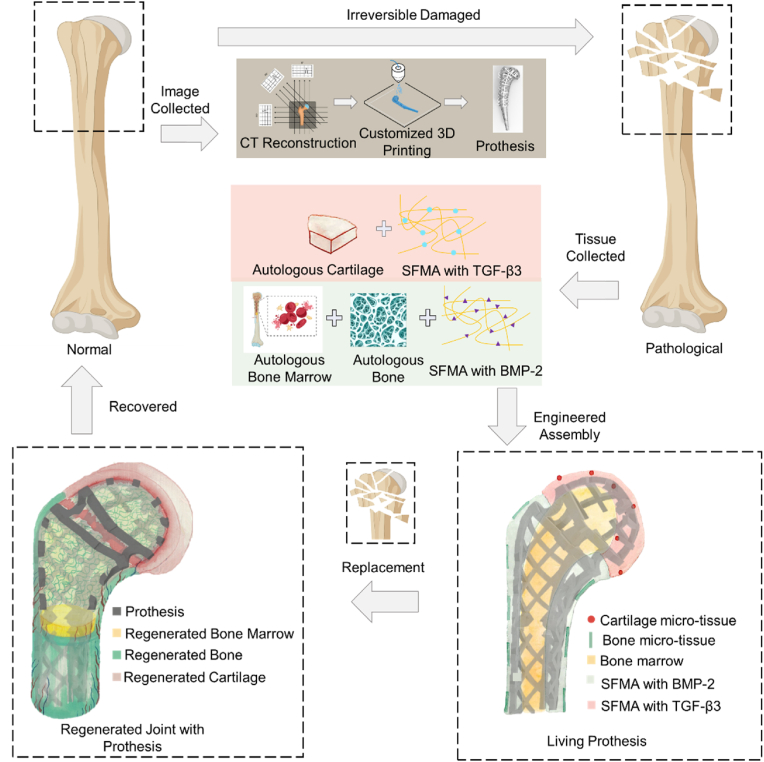
| Schematic diagram of the study design (created with BioRender).

In this study, we introduce a novel concept of a "living prosthesis" that represents a paradigm shift in orthopedic treatments (Scheme 1). This innovative prosthesis not only offers immediate mechanical stability at the defect site but also acts as a guiding scaffold for the precise regeneration of the entire joint. Therefore, living prostheses can achieve the effect of one-time surgery for lifelong use, significantly improving the quality of life of young patients after joint replacement surgery. Specifically, we designed a 3D-printed tower-structured prosthesis that incorporates bone and cartilage units within its intricate grids to foster synergistic regeneration and integration of the metallic prosthesis with the surrounding native tissues. The selection of widely acceptable biomaterials and clinically approved ingredients ensures the translatability of this approach for human joint replacement. This comprehensive strategy ensures the safety of the procedure while enabling the complete regeneration of cartilage, subchondral bone, and bone tissue, ultimately restoring the joint's biological functionality following surgery.

### 1.1 Experimental section

1 Materials

The Mulberry silk was purchased from Zhejiang Xingyue Biotechnology Co., Ltd., Hangzhou, China. GMA was purchased from Sigma-Aldrich (St Louis, MO), Bone Morphogenetic Protein 2 was purchased from Hangzhou Jiuyuan Gene Engineering Co. (China). Transforming Growth Factor-β3(TGF-β3) were purchased from Neobioscience (China). Reagents for cell culture, such as low-glucose medium, high-glucose medium, fetal bovine serum, Penicillin-Streptomycin Solution, Trypsin, Phosphate Buffer Solution, were purchased from Gibico (USA). Cell Counting Kit-8 was purchased from Dojindo (Shanghai, China). Live/dead reagent was purchased from Thermofisher. Alkaline Phosphatase Detection Kit was purchased from Beyotime Biotechnology (Shanghai, China). Alizarin Red S and Alcian Blue were purchased from Sigma-Aldrich (St Louis, MO). Collagen I alpha 1 antibody (NB600-450, at 1:100 dilution) and Collagen II antibody (NB600-844, at 1:100 dilution) were purchased from Novus Biologicals (USA). Fluorescent dye Cy5 was purchased from Solarbio (China). Dexamethasone, Disodium beta-Glycerophosphate Tetrahydrate and Vitamin C were purchased from Aladdin (China).

2 Design and fabrication of 3D printed titanium humeral prosthesis.

Micro-CT scanning was performed on a 3 kg rabbit to obtain the scanned images of the left shoulder joint. The CT image analysis software was then used to reconstruct the three-dimensional image of the left humeral head, and the morphology of the prosthesis was designed based on the reconstructed image. According to previous study, more than 400 μm pore size and more than 50 % porosity can promote bone regeneration [24,25]. Therefore, a grid-like structure with a pore size of 500 μm and 50 % porosity was designed for the main body of the prosthesis. Additionally, pore sizes of 200–300 μm and 80%–90 % porosity can effectively promote cartilage regeneration [26,27]. Hence, a grid-like structure with a pore size of 300 μm was designed for the joint part of the prosthesis. Titanium metal 3D printing was performed by Hunan Huaxiang Medical Technology Co., Ltd. using Selective Laser Sintering Technology. Tantalum metal 3D printing was performed by Ningxia Orient Tantalum Industry Co., Ltd.

3 Cell Viability Assays

Mice bone marrow mesenchymal stem cells (BMSCs) were seeded on Ti2 scaffolds or flat dishes (10 thousand cells per scaffold or flat dish) and incubated at 37 °C, 5 % CO2 and 95 % humidity while the culture medium was refreshed every 2 days. After 1, 3, 5, and 7 days of cultivation, the cell viability was assessed using CCK-8 and a live/dead assay kit in accordance with the manufacturer's instructions. Images of live cells (stained in green by calcein acetoxymethyl) and dead cells (stained in red by propidium iodide) were captured by a fluorescence microscope (DMi8, Leica, Germany).

4 Preparation of Silk Hydrogel

The SFMA was synthesized from silk fibroin (SF) and glycidyl methacrylate(GMA) according to previously study [28]. Briefly, 50 g of sliced cocoon was boiled in 2 L of 0.05 M Na_2_CO_3_ solution for 1 h to remove the sericins and then washed several times with distilled water. Next, the degummed silk was dried at room temperature. Subsequently, 30g of dried degummed silk was dissolved in 150 mL of 9.3 M LiBr solution at 60 °C for 30 min. After completely dissolved, 30 mL of GMA were added to the silk solution slowly and stirred at 300 rpm for 3 h at 60 °C. Then, the mixture solution was collected and dialyzed in 8–14 kDa dialysis tubes (Biosharp) with distilled water for 5 days. Finally, the SFMA solutions were freeze-dried for 72 h, and the SFMA powder was stored at −80 °C for further use.

5 Preparation of Living Prosthesis

SFMA hydrogel was dissolved in PBS (phosphate buffered saline) with concentration of 20 %, A photo-initiator LAP (lithium phenyl-2,4, 6-trimethylbenzoylphosphinate) was added to SFMA hydrogel before use at a final concentration of 0.25 %. Thereafter, bone marrow condensations which were harvested from the autologous rabbit's humerus were mixed into SFMA hydrogel at a final concentration of 10 %, resulting in BM-hydrogel. To this BM-hydrogel, BMP-2 and TGF-β3 were added at final concentrations of 100 μg/mL and 100 ng/mL, respectively, to create an osteogenic and chondrogenic bioactive glue (Table 1). Subsequently, the cartilage from the autologous rabbit's humerus was carefully separated and diced into micro-tissues. These were then mixed with the chondrogenic bioactive glue and used to fill the head of the prosthesis. The cortical bone was divided into bone masses measuring approximately 10 μm in length and 2 μm in width. These bone masses were secured around the stem of the prosthesis and encapsulated with the osteogenic bioactive glue. Upon treatment with UV light (wavelength 488 nm), the living prosthesis was successfully fabricated, which was now ready for implantation, primed to support both bone and cartilage regeneration.

6 The Release Kinetics of Growth Factor from SFMA in Vitro

**Table 1:** | Components of different bioactive glues.

	SFMA (mg/ml)	BMP-2 (μg/ml)	TGF-β3 (ng/ml)
LBMP-SFMA	200	20	0
MBMP-SFMA	200	100	0
HBMP-SFMA	200	500	0
LTGF-SFMA	200	0	20
LTGF-SFMA	200	0	100
LTGF-SFMA	200	0	500
L-SFMA	200	20	20
M-SFMA	200	100	100
H-SFMA	200	500	500

BMP-2 and TGF-β3 were labeled by Cy5, and then encapsulated in 20 % w/v SFMA (the concentration BMP-2 was100 μg/ml, the concentration TGF-β3 was100 ng/ml, n = 5), respectively. Then they were incubated at 37 °C, 5 % CO2 and 95 % humidity with 500 μl PBS. After 1h, 3h, 6h, 12h, 1d, 3d, 7d and 14d of incubation, all PBS were collected and refreshed. The fluence intensity of cy5 in PBS were detected by ELISA (Spark, Tecan, Australia).

7 The Release Kinetics of Growth Factor from SFMA in Vivo

Eight-week-old mice were anesthetized under isoflurane, shaved on both legs, and disinfected with three scrubbing routines of povidone iodine, followed by 70 % (v/v) ethanol. Forty microliters of Cy5 labeled BMP-2, BMP-2/SFMA (the concentration BMP-2 was 100 μg/ml) were injected or implanted into the tibialis anterior muscle. Forty microliters of Cy5 labeled TGF-β3, TFG-β3/SFMA (the concentration TGF-β3 was 100 ng/ml) were injected or implanted into the joint capsule. An in vivo imaging system (IVIS, Optima, Biopace, America) and Living Image software were used to serially acquire and quantify fluorescence over a period of 7 days. 7 or 14 days after the implantation, mice were sacrificed and the hydrogel were collected and then detect the fluence intensity of cy5 by IVIS.

8 In Vitro Analysis of Osteogenic Differentiation

To evaluate the efficiency of BMP-2 in the modulation of osteogenic differentiation in vitro, 20 μg/ml, 100 μg/ml, 500 μg/ml of BMP-2 encapsulated in 20%w/v SFMA were selected. One million per milliliter mice BMSCs were seeded on the surface of SFMA, LBMP-SFMA (20 μg/ml BMP-2), MBMP-SFMA (100 μg/ml BMP-2) and HBMP-SFMA (500 μg/ml BMP-2) (n = 10). The osteogenic medium (Table 2) were refreshed every 2 days. Seven days later, the hydrogels were collected and fixed by paraformaldehyde, and then evaluated the efficiency of osteogenic differentiation by alizarin red staining and alkaline phosphatase (ALP) staining.

9 In Vitro Analysis of Chondrogenic Differentiation

**Table 2:** | Components of osteogenic medium.(10 ml).

Components	Volume	Final Concentration
H-DMEM	8.7 ml	/
Foetal Bovine Serum	1 ml	10 %
Disodium beta-Glycerophosphate Tetrahydrate	100 μl	10 mmol/L
Dexamethasone	100 μl	10-8M
Vitamin C	100 μl	50 μg/ml

To evaluate the efficiency of TGF-β3 in the modulation of chondrogenic differentiation in vitro, 20 ng/ml, 100 ng/ml, 500 ng/ml of TGF-β3 encapsulated in 20%w/v SFMA were selected. 1 million/ml mice BMSCs were seeded on the surface of SFMA, LTGF-SFMA (20 ng/ml TGF-β3), MTGF-SFMA (100 ng/ml TGF-β3) and HTGF-SFMA (500 ng/ml TGF-β3) (n = 5). The chondrogenic medium (Table 3) were refreshed every 2 days. 14 days later, the hydrogels were collected and fixed by paraformaldehyde, and then evaluated the efficiency of chondrogenic differentiation by alcian blue staining.

10 The local and systemic immune responses of bioactive glues in vivo.

**Table 3:** | Components of chondrogenic medium.(10 ml).

Components	Volume	Final Concentration
H-DMEM	8.6 ml	
Dexamethasone	100 μl	10-7M
Vitamin C	100 μl	50 μg/ml
ITS	100 μl	1 %
Sodium pyruvate	100 μl	1 mM

In this project, 50 WT mice (strain C57BL/6JNifdc, 2 months age) were collected from animal center in Haining Campus, Zhejiang University. hey were treated under standard animal ethic guidelines approved by the Institutional Animal Care and Use Committee, Zhejiang University (ZJU20241106). They were divided into 5 groups (n = 10), Sham group, SFMA group, L-SFMA group (200 μl 20%w/v SFMA with 20 μg/ml BMP-2 and 20 ng/ml TGF-β3), M-SFMA (200 μl 20%w/v SFMA with 100 μg/ml BMP-2 and 100 ng/ml TGF-β3), H-SFMA (200 μl 20%w/v SFMA with 500 μg/ml BMP-2 and 500 ng/ml TGF-β3).

Mice were anesthetized by isoflurane and the back were shaved and disinfected with povidone iodine scrub, followed by 70 % (v/v) ethanol in water. Then they were transferred to a heated operating table to keep warm and draped in a sterile manner. An incision was made with a #11 scalpel to expose the subcutaneous tissue and the hydrogels were implanted into the subcutaneous tissue. The skin was closed with 6-0 sutures and antibiotics were administered by intramuscular injection to prevent infection. 7 and 14 days later, the skins, organs and blood serums were collected to evaluate the local and systemic immune response.

11 Left Humerus Resection and Joint Replacement Surgery in Rabbits

In this project, 36 News Zealand rabbits (male, 3 Kg in average) were collected from animal center in Zijingang Campus, Zhejiang University. They were treated under standard animal ethic guidelines approved by the Institutional Animal Care and Use Committee, Zhejiang University (ZJU20210247). Rabbits were anesthetized with intravenous Zoletil 50 (10 mg/kg) and pentobarbital (20 mg/kg). A total of 36 male New Zealand white rabbits were randomly divided into three groups (n = 12 per group): autologous bone devitalization and replantation group (clinical gold standard group) (ABG group), blank prosthesis group (BP group), living prosthesis group (LP group). Half an hour before the surgery, buprenorphine patch was attached to the back skin to relieve the pain during the joint replacement surgery. After anesthesia, an approximately 4 cm incision was made along the shoulder joint's outer side and expose the proximal segment of the humeral shaft. Then cut down the ligament around the humeral head. Next, we can separate the humerus from the greater tubercle. For ABG group, the humerus shaft and joint were boiled in 0.9 % sodium chloride for 30 min at 100 °C and then put the humerus back with steel plates to fix, 3-0 PROLENE sutures to suture the joint capsule. For BP group, the scaffold was inserted into the medullary cavity of the humerus and fixed with screws and the joint capsule was fixed on the scaffold. For LP group, the prepared living prosthesis was fixed as the BP group. Finally, closed the wound with 4-0 sutures and antibiotics were administered by intramuscular injection to prevent infection. Postoperatively, the rabbits were allowed to move freely in their cages and were fed standard food and water. Meloxicam was fed for 3 days to relieve the pain after operation. The motor function was evaluated at 2 months postoperatively.

12 X-ray and Micro-CT Evaluation

At one, four- and eight-weeks post-operation, the rabbits were anesthetized with pentobarbital and placed on an X-ray machine (Zhejiang University Animal Hospital) to assess bone regeneration. Eight weeks after operation, the rabbits were euthanized with overdose of pentobarbital. The entire humerus, including the joint capsule and tendons attached to the humeral head were harvested. Samples (n = 3 per group) were scanned by small animal in vivo Micro Computed Tomography (Netherland, Milabs) and subsequently reconstructed.

13 Biomechanical Analysis

The collected samples were subjected to biomechanical testing using a Universal Testing Machine (MTS). This included three-point bending and prosthesis pull-out experiments to evaluate the mechanical properties of the regenerated bone tissue.

14 Histological Analysis

A portion of the collected samples was processed for hard tissue sectioning. Collected samples with the prosthesis in place were fixed in 4 % PFA for 2 days followed by a rinse in distilled water and then stored in 70 % ethanol till processing. The samples were dehydrated with a gradient of ethanol (70 %, 80 %, 90 %, 95 %, 100 %) and then embedded in Methyl Methacrylate (MMA). Slices with a thickness of approximately 300 μm were obtained using the EBAKT-300CP slicer and stained with Hematoxylin and Eosin (H&E), Safranin O/Fast Green (SO/FG), and Masson staining to determine the degree of development of newly formed bone and cartilage. The newly formed bone and joint were separated from the implant, decalcified with EDTA, embedded in paraffin, and sectioned for staining to observe the regenerative effects of the newly formed bone and cartilage tissues.

15 Statistical Analysis

All statistical analyses were performed using Prism. A significance level of p < 0.05 was set for statistical significance. One-way analysis of variance (ANOVA) was used for statistical analysis, followed by post-hoc tests, such as the least significant difference (LSD) test, to compare selected data pairs.

## 2 Results

1. Design of a light-weight “living prosthesis” with excellent mechanical strength.

**Fig. 1:**
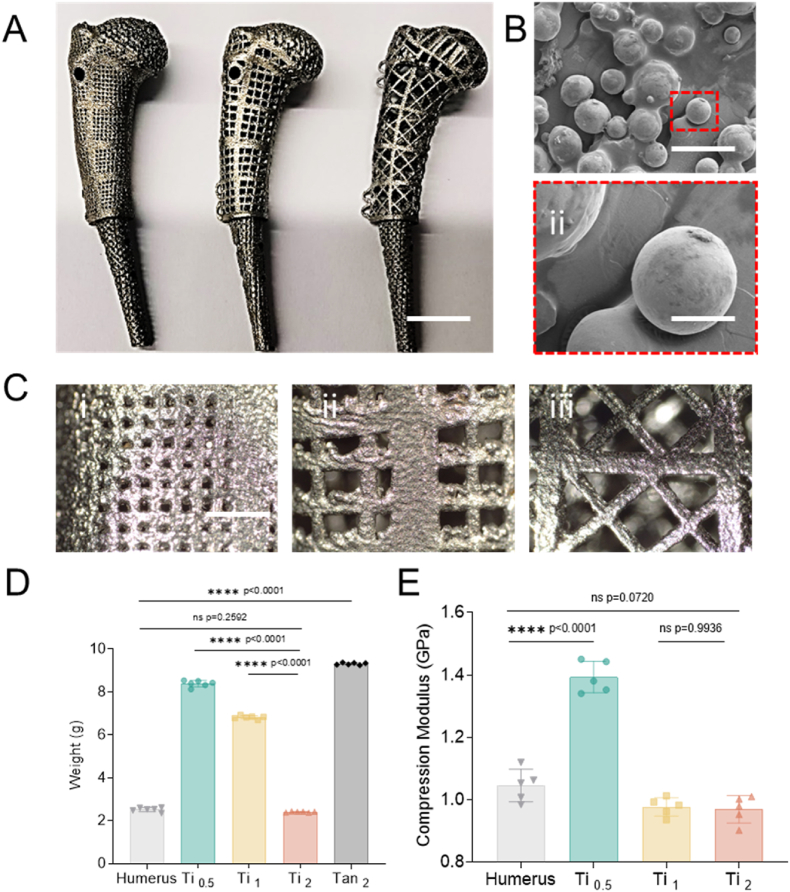
**| The characterization of rabbit humerus titanium scaffold.** (A) The morphology of Ti Scaffold with different pore size. Scale bar represents 1 cm. (B)The SEM of the surface of Ti_2_ Scaffold, (i) 500X, Scale bar represents 50 μm.(ii)2000X. Scale bar represents 10 μm. (C) The focus image of Ti_0.5_(i), Ti_1_(ii), Ti_2_(iii). Scale bar represents 2 mm. (D) The weight of normal humerus, Ti_0.5_, Ti_1_, Ti_2_ and Tan_2_. (E) The Modulus of Ti_0.5_, Ti_1_and Ti_2_. Significant differences are presented, exact p-value was calculated with one-way ANOVA Tukey's multiple comparison test.

To achieve whole-joint regeneration, we aimed to mimic both the structure and mechanical properties of the original joints. Thus, the prosthesis was designed on the basis of CT scans of a rabbit's left humerus and constructed from titanium through selective laser melting (SLM) 3D printing technology (Fig. 1A). This prosthesis consists of three distinct regions: an ellipsoidal (mushroom-like) grid structure at the top (16 mm × 12 mm × 10 mm), a cylindrical porous structure in the middle (8.5 mm × 7 mm × 20 mm), and a bottom insertion stem for surgical fixation (4.5 mm × 3.4 mm × 20 mm) (Fig. 1B). To optimize the weights and strengths of the prostheses, we printed three types of prostheses with increasing pore sizes and porosities: Ti_0.5_, Ti_1_ and Ti_2_ (Fig. C, S1A). The weights of the Ti_2_ prostheses (2.405 ± 0.022 g) were similar to those of a normal humerus (2.513 ± 0.090 g) and significantly lower than those of both the Ti_0.5_ (8.380 ± 0.135 g) and Ti_1_ (6.810 ± 0.070 g) prostheses (Fig. 1D and E). Compared with tantalum protheses, another widely used metal material in clinical medicine [29], Ti_2_ protheses are approximately one-quarter the weight of tantalum protheses (2.405 ± 0.022 vs 9.306 ± 0.046 g). Despite being lightweight, the compression modulus of the Ti_2_ prosthesis was comparable to that of a normal humerus (0.969 ± 0.002 GPa vs 1.045 ± 0.003 GPa) and only slightly lower than that of Ti_0.5_ (1.393 ± 0.002 GPa). Therefore, Ti_2_ protheses were selected for further studies because of their excellent mechanical properties. To investigate the biological influences of these protheses, scanning electron (SEM) was used to characterize the surface morphology of the samples, revealing the relatively rough surface of the Ti_2_ prosthesis, which can promote tissue adhesion and integration [30,31] (Fig. 1B). The excellent biocompatibility of the titanium prosthesis was further confirmed by culturing bone marrow-derived mesenchymal stem cells (BMSCs) on the Ti_2_ prosthesis (Figs. S2A and 2B).

To guide the correct tissue regeneration in the corresponding joint regions as well as to achieve the tight integration between the living prosthesis and regenerated tissues, biologically active glues compromised with silk fibroin hydrogel loaded with osteogenic and chondrogenic growth factors were applied on the bone stem and humeral head region, respectively. On the basis of our previous research, silk fibroin-methacrylate (SFMA) is selected as a suitable biomaterial with great biocompatibility, mechanical properties and can promote the regeneration of joints[[19], [20], [21]]. As growth factors play key roles in regulating the correct tissue regeneration, transforming growth factor-beta 3 (TGF-β3) and bone morphogenetic protein-2 (BMP-2) are chose to promote cartilage and bone generation [22,23].

To explore the release kinetics of growth factors encapsulated by SFMA, we conducted an in vitro release experiment of growth factors (Fig. S3A). The release profiles for both BMP-2 and TGF-β3 exhibited similar patterns. Specifically, at the first day, BMP-2 was released at 51.69 % ± 2.4 %, and TGF-β3 at 53.22 % ± 2.4 %. Subsequently, the release rate slowed down, with 74.31 % ± 3.78 % of BMP-2 and 73.12 % ± 3.89 % of TGF-β3 released by day 14, respectively (Figs. S3B–C). To validate the retention of these growth factors in vivo, we implanted fluorescently labeled BMP-2 and TGF-β3 into muscle and knee joint tissues. Using an in vivo imaging system (IVIS), we serially measured the fluorescence within these tissues over a seven-day period (Fig. S4A). Three hours post-implantation, free BMP-2 and TGF-β3 were barely detectable, whereas BMP-2/SFMA and TGF-β3/SFMA showed strong signals that persisted for up to one week (Figs. S4B–C). These findings align with the in vitro results (Figure S4F-G, S4I-J), indicating that SFMA significantly prolongs the retention of BMP-2 and TGF-β3 in vivo. Then we collected the hydrogels at day 7 and 14 post-implantation. Using IVIS, we confirmed that BMP-2 and TGF-β3 could still be detected until day 14 (Figure S4D-E, S4H-K). These results demonstrate that SFMA can gradually release BMP-2 and TGF-β3 over a period of up to 14 days.

In vitro and in vivo experiments were conducted using low, middle and high concentration of TGF-β3 and BMP2 to determine the optimal composition of bioactive glue. Alizarin red and alkaline phosphatase staining demonstrated that, compared with bare SFMA, SFMA with BMP-2 effectively facilitated bone regeneration. Besides, there was no significant difference between MBMP-SFMA and HBMP-SFMA (Fig S5A-B,5D-E) (Table 1). Alcian blue staining demonstrated that, compared with bare SFMA, SFMA with TGF-β3 can effectively facilitated cartilage regeneration and similar chondrogenic ability between MTGF-SFMA and HTGF-SFMA (Figs. S5C and 5F).

To evaluate the biocompatibility of our bioactive glues by implanting SFMA loaded with various concentrations of growth factors into the dorsal region of mice. Hematological and biochemical analyses, as well as histological staining of skin, organs, and serum, were performed at 3 and 14 days postoperatively. At 3 days post-implantation, a transient inflammatory response was observed around the SFMA, which is a normal immune reaction to a foreign material (Fig. S6A). By day 14, the inflammatory cells had disappeared in the SFMA, L-SFMA, and M-SFMA groups, indicating excellent biocompatibility of these hydrogels. However, in the H-SFMA group, inflammatory cell aggregation was still evident (Fig. S6B), suggesting that high concentrations of growth factors may cause local tissue irritation. Biochemical parameters and organ histological staining showed no significant systemic toxicity in any group at either the short-term (3 days) or longer-term (14 days) time points (Figs. S7 and S8).

Above all, 100 μg/ml BMP-2 and 100 ng/ml TGF-β3 were the suitable concentration of growth factor to promote the joint regeneration.

2. Radiological evaluation validated the well-regenerated bone in the “living prosthesis”.

We conducted a rabbit humerus hemi-resection, followed by replacement of the living prosthesis with biological glues. Serial X-ray imaging revealed the regeneration of bone tissue around the prosthesis, ultimately leading to a functionally active living prosthesis joint (Fig. S1C).

Thirty-six rabbits underwent left humerus resection. Twelve patients were treated with autologous bone graft (ABG) surgery, and twenty-four patients were treated with joint replacements, with twelve of whom receiving blank prosthesis (BP) and twelve receiving living prostheses (LP). All rabbits regained normal motor functions after surgery. X-ray follow-ups were performed at one week, four weeks, and eight weeks post-operation. When X-rays revealed full regeneration of the bone defect, the rabbits were sacrificed for further evaluation, including micro-CT for radiological assessment, mechanical testing for functional evaluation and histological analysis for tissue regeneration evaluation (Fig. S9).

**Fig. 2:**
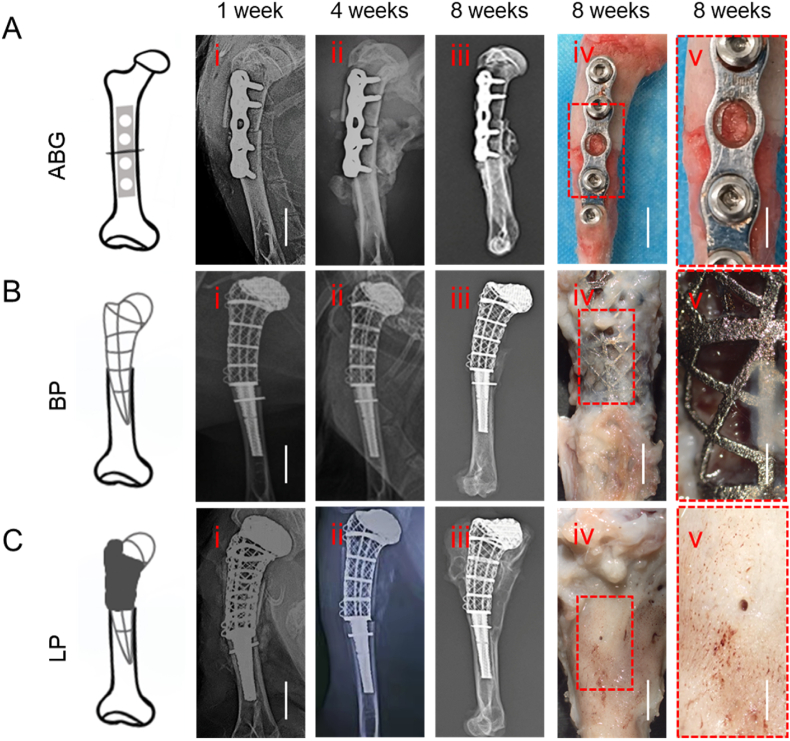
**| Living prosthesis showed intact morphology and structure of cortical bone**. The general evaluation of transplanted humerus in 1, 4 and 8 weeks post-surgery. The X-ray follow-up of transplanted humerus in 1(i), 2(ii), 8(iii) weeks post-surgery (Scale bar represents 1 cm) and gross appearance of that in 8 weeks (iv, v) (iv. Scale bar represents 5 mm, v. Scale bar represents 2 mm) post-surgery in ABG group(A), BP group (B) and LP group(C).

One-week post-operation, all the prostheses and fixations were securely in place, with no significant displacement or joint dislocation (Fig. 2A(i), 2B**(i), 2C(i)**). Four weeks post-surgery, there was no obvious callus between the bone cracks in the ABG group, and the BP group presented little neoformation. In contrast, the LP group presented some osteoid formation around the prosthesis (Fig. 2A(ii), 2B(i**i), 2C(ii)**). Eight weeks post-surgery, the LP group exhibited regeneration of cortical bone around the entire prosthesis, whereas the neo-ossification in the BP group was located mainly at the bottom of the scaffold. In the ABG group, the cortical bone between the replanted bone and the original bone was still discontinuous, and the density of the replanted bone and the proximal end of the humerus was significantly lower on X-ray, indicating possible bone destruction and resorption (Fig. 2A(iii), 2B(iii**), 2C(iii)**). Gross images of the samples collected eight weeks after surgery revealed fibrous tissue filling the bone cracks in the ABG group, which might have resulted in weaker mechanical function. In the BP group, fibrous tissues adhered to the prosthesis surface, with no obvious regenerated bone tissue on or inside the scaffolds. In the LP group, most of the new cortical bones were completely regenerated, with visible capillary networks on the surface (Fig. 2A (iv,v), 2B (iv,v), 2C (iv,v)).

**Fig. 3:**
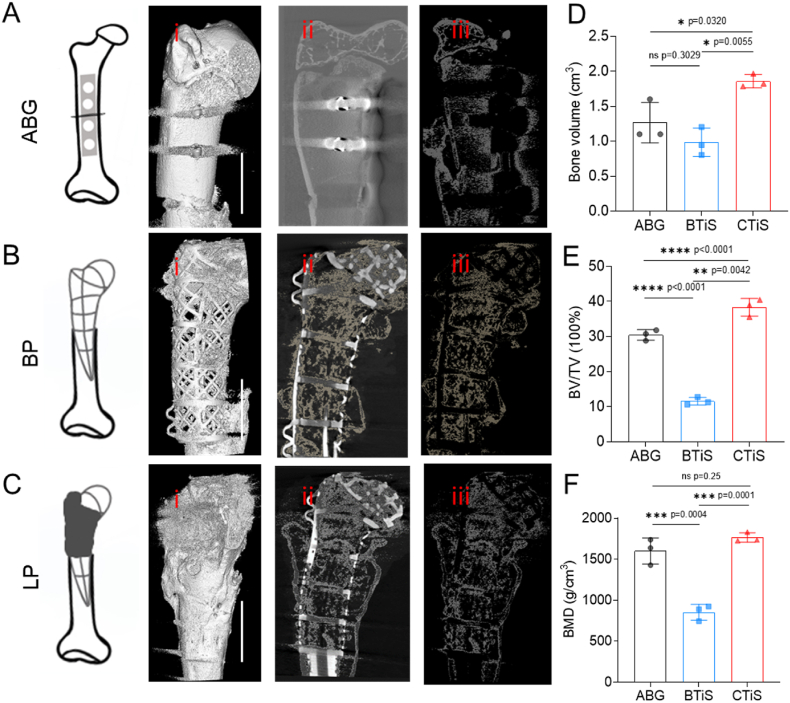
**| Increased new bone formation in living prosthesis 8 weeks after transplantation.** Micro-CT images of ABG group (A), BP group (B) and LP group (C). Living prosthesis showed similar bone volume (D), bone value/total value (E) and bone mineral density (F) to autologous bone graft. Scale bar represents 1 cm. Significant differences are presented, exact p-value was calculated with one-way ANOVA Tukey's multiple comparison test.

Micro-CT analysis was used to analyze the quality of newborn bone accurately. Similar to the X-ray findings, 3D reconstruction revealed poor repair between the autologous bone graft and the native cortical bone in the ABG group, indicating incomplete cortical bone continuity (Fig. 3A (i, ii, iii)). In the BP group, low-density tissues were affiliated with the surface of the scaffold rather than the osteoid surface (Fig. 3B (i, ii, iii)). In the LP group, the scaffolds were enclosed by new bone (Fig. 3C (i, ii, iii)). Compared with the BP group, the LP group presented greater bone volume, a greater bone volume/total volume ratio and greater bone mineral density (1.859 ± 0.096 vs 0.985 ± 0.203 cm^3^, 38.35 % ± 2.51 % vs 11.58 % ± 1.08 %, 1766.14 ± 58.01 vs 855.16 ± 95.60 g/cm^3^), similar to those of the ABG group, which is the gold standard treatment for bone defects (1.267 ± 0.289 cm^3^, 30.46 % ± 1.51 %, 1602.55 ± 158.02 g/cm^3^) (Fig. 3D, E, 3F).

3. The “living prosthesis” achieved full joint restoration with precise regeneration of the osteochondral tissues.

**Fig. 4:**
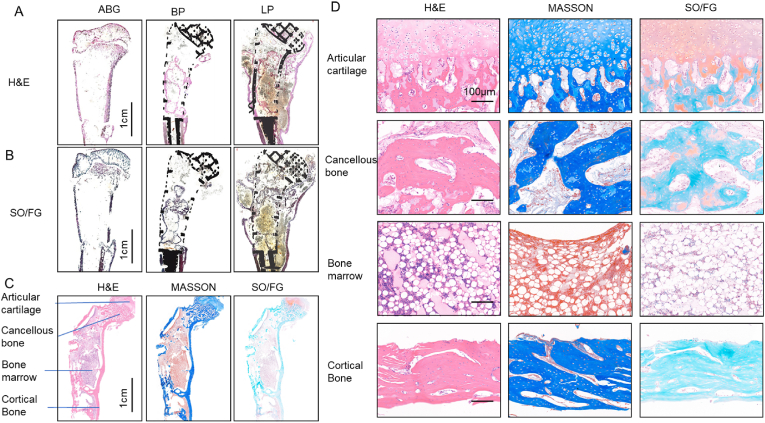
**| Correct regeneration of corresponding tissues around the living prosthesis.** Hematoxylin-Eosin Staining(H&E) (A) and Safarin O/Fast green staining (SO/FG) (B) of hard tissue section with ABG, BP and LP group. (C) Cartilage, cancellous bone, bone marrow and cortical bone regeneration evaluated by H&E, Masson Staining and SO/FG. (D) Magnification of cartilage, cancellous bone, bone marrow and cortical bone in(C). Scale bar represents 100 μm.

To further evaluate the regeneration outcomes, the total joints along with the prostheses were harvested and subjected to hard tissue sectioning (Fig. 4A). Consistent with the radiology results, the cortical bone in the ABG group remained discontinuous, with a clear gap between the transplanted grafts and injury sites. Notable loss of the cartilage matrix in the articular cartilage was also found in the ABG group. The BP group presented minimal fibrous tissue formation with the prosthesis. Remarkably, the LP group presented newly formed cortical bone surrounding the prosthesis and bone marrow-like tissues filling the interior (Fig. 4B).

To systematically evaluate the regenerated tissues in the LP group, the newly formed cortical bone and cartilage were separated from the prosthesis for detailed histological analysis (Fig. 4C). The results revealed highly organized and precise regeneration of joint tissues at various sites. Hematoxylin‒eosin (H&E) staining revealed that the cells in the articular cartilage layer presented a distinct chondrocyte-like morphology, with cells arranged in pairs within well-defined cartilage lacunae. In the subchondral bone and cortical bone of the diaphysis, the cells were spindle shaped and eccentrically located in the nucleus. The subchondral bone displayed a trabecular structure arranged in a load-bearing manner, while the cortical bone trabeculae were tightly packed. The bone marrow cavity contains abundant lipid droplets and hematopoietic cells. Masson staining confirmed that both the subchondral bone and cortical bone of the diaphysis are composed of newly formed bone tissue, as evidenced by intense blue staining. Safranin O/fast green (SO/FG) staining further validated the presence of cartilage-specific glycosaminoglycans in the articular cartilage region, whereas the subchondral and cortical bone were stained with fast green. Furthermore, our immunofluorescence analysis of the regenerated tissue in the LP group revealed that the tissues surrounding the prosthesis head exhibited a high expression of collagen Ⅱ, whereas the tissues adjacent to the prosthesis stem demonstrated a prominent expression of collagen I. (Fig. S10). Raman spectroscopy analysis confirmed the presence of abundant phosphate ions in the regenerated tissue of the LP group, which are essential for the formation and maintenance of the bone matrix (Fig. S11).

To assess the functional integrity of the regenerated osteochondral tissues, we collected glenoid cavity samples from different groups and evaluated the wear conditions. In the ABG group, the articular cartilage of the humeral joint and glenoid cavity remained relatively intact, with minimal wear and tear, as evidenced by H&E and SO staining (Figs. S12A, S12D, S12G). In contrast, the BP group exhibited direct contact and friction between the prosthesis and glenoid cavity, resulting in severe cartilage damage extending to the subchondral bone (Figs. S12B, S12E, S12H). In the LP group, the majority of the prosthesis surface was covered by regenerated cartilage-like tissue, with only moderate loss of cartilage extracellular matrix in the glenoid cavity (Figs. S12C, S12F, S12I). And the cartilage OARSI score [32] and light microscope mankin score [33] demonstrated the LP group can effectively protect the contralateral cartilage (Figs. S12J and S12K).

4. Mechanical evaluation revealed superior anti-fracture properties, resistance to tendon avulsion and resistance to prosthesis dislocation in “living prostheses”.

**Fig. 5:**
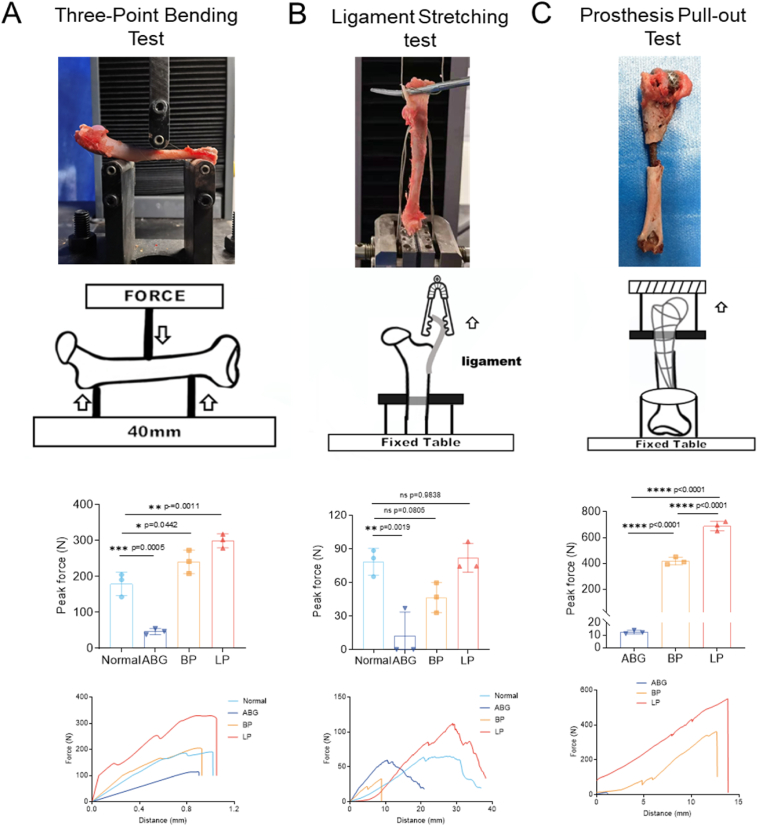
**| Living prosthesis were successfully integrated into native tissue and exhibited great mechanical performance.** (A) Three-point bending test, (B) Ligament stretching test, (C) Prosthesis pull-out test of transplanted humerus in 8 weeks post-surgery. Significant differences are presented, exact p-value was calculated with one-way ANOVA Tukey's multiple comparison test.

To investigate the mechanical functionality of the regenerated joints, three biomechanical tests were conducted. First, three-point bending tests were performed to evaluate the ability of the humerus to resist transverse fractures. Second, ligament stretching tests were conducted to measure the tensile resistance of the regenerated joint capsule. Finally, prosthesis pull-out tests were used to test the tensile resistance of the prosthesis. The LP group exhibited an impressive anti-fracture force of 299.6 ± 16.0 N, which significantly exceeded those of the BP group (241.0 ± 26.5 N) and the normal humerus group (179.7 ± 26.5 N). In contrast, the ABG group, due to bone nonunion, could withstand an anti-fracture force of only 46.7 ± 6.9 N (Fig. 5A). Ligament stretching tests revealed superior strength in the LP group (82.0 ± 10.4 N), comparable to that in the normal joint capsule (78.5 ± 9.7 N), and significantly greater than that in the BP group (46.5 ± 11.0 N) and ABG group (12.3 ± 17.4 N), indicating improved integration and tear resistance in the LP group (Fig. 5B). In addition, the LP group exhibited outstanding anti-pull-out mechanical ability (689.8 ± 29.9 N) compared with the BP group (417.8 ± 23.9 N) and ABG group (12.4 ± 1.2 N). These results highlight the excellent performance of the LP group in resisting transverse fractures, longitudinal stretching, and ligament pull-out.

The locomotor ability of rabbits in the LP group was significantly restored at two months post-surgery. Rabbits in the LP group exhibited superior rotational activities and jumping abilities, closely resembling near-normal levels (Movie S1). Additionally, histological examination of major organs via HE staining revealed no obvious abnormal pathological changes, such as fibrosis, hemorrhage, or apoptosis, in any of the groups, indicating the safety of the living prosthesis strategy (Fig. S13).

## 3 Discussion

Despite advancements in tissue engineering techniques for regenerating mild to moderate tissue defects, the total replacement of osteoarticular defects still serves as the preferred option for severe-grade joint injuries, such as late-stage osteoarthritis and bone neoplasms[[34], [35], [36], [37], [38]]. However, current joint prostheses primarily offer mechanical support with limited biological functionality, and the incongruity between the prosthesis and native bone can often precipitate issues such as prosthetic loosening and fractures in the years following surgery. Individuals may require multiple total joint replacement surgeries over time, with each subsequent procedure typically involving greater pain, blood loss, and financial cost. To address these limitations, we introduced an innovative "living prosthesis" strategy that provides one-time surgery for lifelong use, increasing the quality of life for younger patients after joint replacement surgery.

In our study, we successfully demonstrated the precise regeneration and seamless integration of various anatomical components, including hyaline cartilage, subchondral bone, cortical bone, the bone marrow cavity, and tendon-bone enthesis, by utilizing a pro-regenerative prosthesis and harnessing the adjacent tissues surrounding the defect sites. Notably, only fractured bone and cartilage fragments, which are typically discarded in traditional joint replacement surgeries, are incorporated into living prostheses, thereby enhancing the natural processes of tissue regeneration. This approach ensures the restoration of functional joints while maximizing the preservation of local tissues and eliminating the risk for secondary damage to donor sites.

To evaluate the efficacy of our living prosthesis, we constructed a rabbit model for upper limb defects, which allows for better control of postoperative exercise levels, which is crucial for optimal recovery. Compared with the lower limb regeneration model, the low-load exercise model of upper limb regeneration has a lower degree of tissue regeneration disorders caused by excessive postoperative exercise [39]. By creating an extremely large defect model combining total joint and large-segment bone defects, we introduced significant regenerative challenges not commonly observed in other models. The utilization of 3D-printed hollow prostheses presents a promising platform for guiding tissue regeneration, minimizing the dependency on graft tissues. Titanium-based prostheses effectively meet the immediate mechanical demands of the replaced joint post-surgery. Through refinement of the structural design of the titanium scaffold, we achieved mechanical harmony with neighboring bone tissue, which can effectively minimize stress shielding to avoid osteoporosis and evoke precise osteogenic and mechano-biological responses to promote bone and cartilage ingrowth [16]. Additionally, the lightweight scaffold design alleviates postsurgical discomfort by closely replicating the weight of natural humeral joints.

One of the major challenges in osteochondral regeneration is the successful regeneration of cartilage tissue because of its avascular nature and limited regenerative capacity [[40], [41], [42]]. Notably, our living prosthesis regenerated whole joints, including the critical cartilage component. Cartilage is crucial for joint function, providing lubrication and stress distribution [43,44]. To promote the regeneration of cartilage, silk fibroin and TGF-β3 were used. Silk fibroin (SF) exhibits excellent biocompatibility and mechanical flexibility, making it a superior material for repairing hard tissues such as cartilage [45,46]. Furthermore, the TGF-β family is widely used to promote cartilage regeneration by recruiting progenitor cells and inducing chondrogenesis [23,47,48].

Another significant limitation of current joint prostheses is their limited lifespan, as they often require revision surgeries due to prosthesis loosening, periprosthetic fractures, infection and prosthesis collapse[[49], [50], [51], [52]]. Revision surgeries present challenges such as tissue adhesions, which can impede the precise separation of vital blood vessels and nerves; stress shielding-induced osteoporosis, which hinders the effective fixation of new prostheses; and mismatches between available prostheses and excessive defect areas. Consequently, joint replacement surgeries for young patients are fraught with significant obstacles [53]. Our living prosthesis has mechanical properties similar to those of normal cortical bone. Two months post-implantation, the mechanical properties of the active prosthesis are comparable to those of native tissue, greatly reducing the frequency of periprosthetic fractures and osteoporosis. Additionally, the newly formed bone and cartilage are well integrated with the prosthesis, making it a part of the body and helping reduce the prosthesis loosening rate. One limitation of our prosthesis model is its design, which is based precisely on the size of the humeral bone of 3kg weight rabbits, leading to an enlarged regenerated humeral bone compared with the native bone. To overcome this problem, the volume of regenerated tissues should be taken into consideration, and a slimmer version of living prosthesis should be designed.

In conclusion, our study introduces a "living prosthesis" strategy for addressing extensive osteoarticular defects in clinical settings. This innovative living prosthesis offers immediate mechanical support and facilitates tissue regeneration via the use of a small amount of graft tissue within a short timeframe. The materials utilized, including titanium and silk fibroin, are well established in orthopedic research, underscoring the potential for seamless integration into existing medical protocols. Our research not only highlights substantial restorative effects for complex joint defects but also holds tremendous promise for practical clinical translation and application.

## CRediT authorship contribution statement

**Wei Sun:** Writing – original draft, Validation, Project administration, Methodology, Investigation, Formal analysis, Data curation, Conceptualization. **Hongwei Wu:** Writing – review & editing, Supervision, Project administration, Investigation, Funding acquisition, Conceptualization. **Yiyang Yan:** Writing – original draft, Methodology, Investigation, Formal analysis, Data curation, Conceptualization. **Xianzhu Zhang:** Writing – review & editing, Project administration, Methodology, Investigation, Funding acquisition. **Xudong Yao:** Writing – review & editing, Investigation. **Rui Li:** Methodology, Investigation. **Jingyi Zuo:** Methodology. **Wenyue Li:** Formal analysis. **Hongwei Ouyang:** Writing – review & editing, Supervision, Project administration, Investigation, Funding acquisition, Conceptualization.

## Ethics approval and consent to participate

The animal study was approved by the Institutional Animal Care and Use Committee, Zhejiang University (ZJU20210247, ZJU20241106).

## Data availability

The data that support the findings of this study are available from the corresponding author upon reasonable request.

## Declaration of competing interest

The authors declare no conflict of interest.
