# Supplementary material for: Living joint prosthesis with in-situ tissue engineering for real-time and long-term osteoarticular reconstruction
Source: Bioact Mater. 2025 Feb 26;48:431–42. doi: 10.1016/j.bioactmat.2025.01.036 (PMC11908457; doi:10.1016/j.bioactmat.2025.01.036)
Supplement: Multimedia component 2 [file mmc2.docx]

**Supplementary Information**


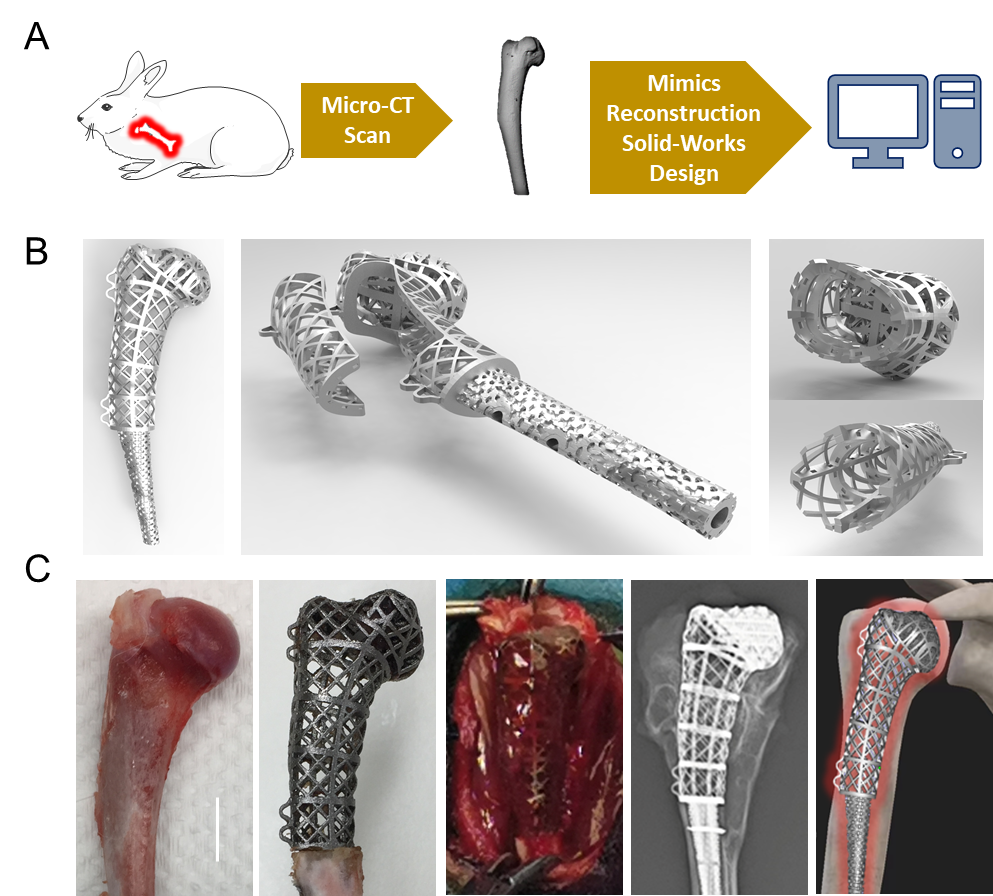


**Figure S1 | The design of the living prosthesis for rabbit humorous joint replacement.** (A) The design of titanium scaffold from the CT scan of rabbit humerus. (B) The designed structure of humerus titanium scaffold. (C) The procedure of the implantation and regeneration of the Living Ti Scaffold. Scale bar represents 1 cm.


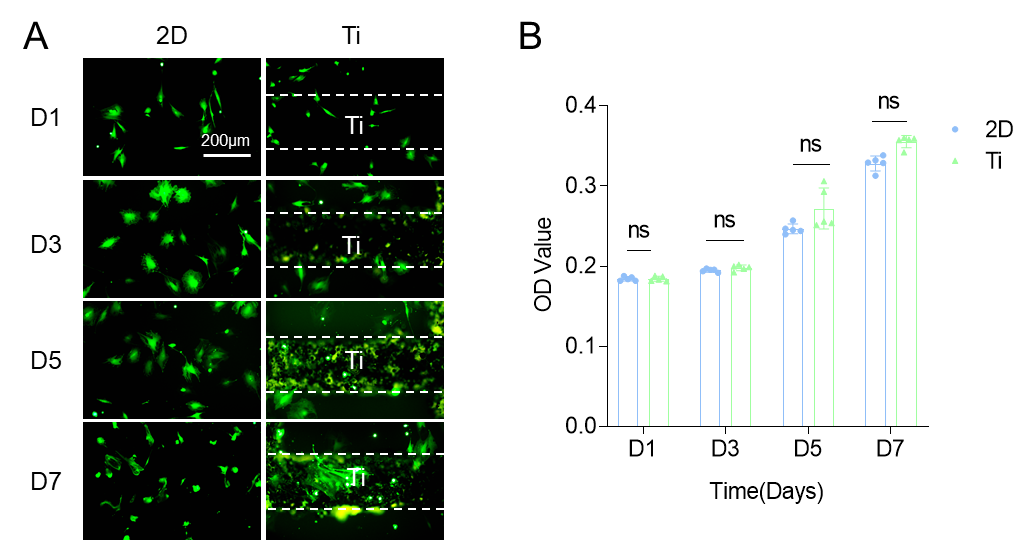


**Figure S2 | The biocompatibility of Ti_2_ scaffold.** (A) The live/dead staining of rabbit BMSCs cultured with Ti_2_ scaffold at 1,3,5 and 7days. Scale bar represents 200μm. (B) CCK-8 assay showed the proliferation of cells in the 2D culture dish and Ti_2_ scaffold at 1,3,5 and 7days. Significant differences are presented, exact p-value was calculated with two-tailed student’s t-tests.


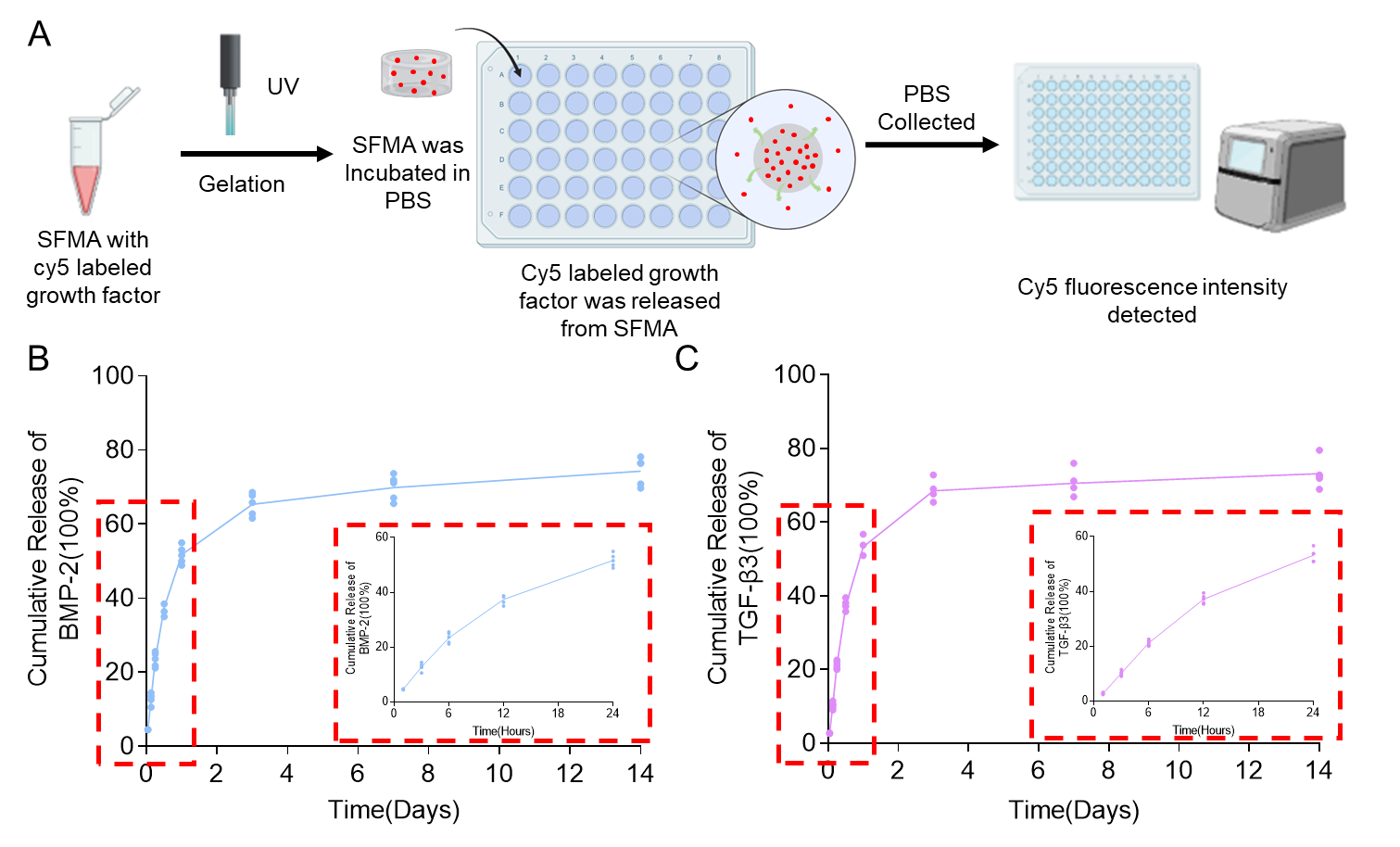


**Figure S3 | The BMP-2 and TGF-β3 were gradually released from SFMA.** (A) The schematic diagram of growth factor release assay. (B) The accumulated BMP-2 release over 14 days from SFMA. (C) The accumulated TGF-β3 release over 14 days from SFMA.


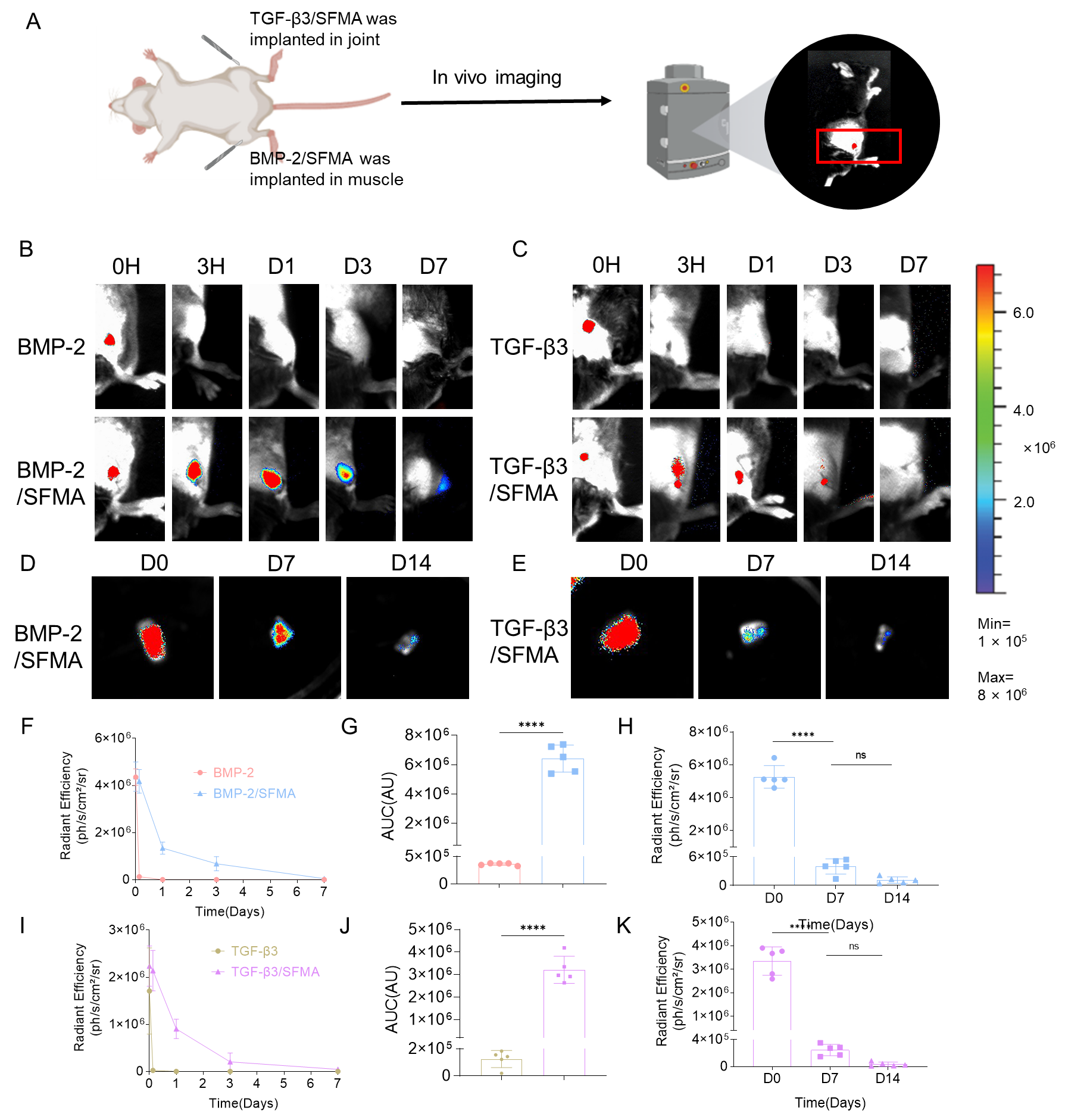


**Figure S4 | SFMA extend the residence of growth factor in vivo.** (A) Schematic of IVIS image with mice muscle BMP-2/SFMA implanted or joint TGF-β3/SFMA. The field of view is shown in the red box. Representative IVIS images of mice legs over 7 days after implanted of fluorescent BMP-2 formulations(B) and TGF-β3 formulations(C). Representative IVIS images of BMP-2/SFMA harvest from mice 7 days(D) and 14 days(E) post-surgery. Time course of fluorescent radiant efficiency of BMP-2(F) and TGF-β3(I) in vivo. Data are fit to a one-phase exponential decay with a common plateau based on background signal. Area under the curve (AUC) results for the BMP-2(G) and TGF-β3(J) retention. Quantitative analysis of fluorescent radiant efficiency of BMP-2/SFMA(H) and TGF-β3(K) harvest from mice 7 and 14 days post surgery. Significant differences are presented, exact p-value was calculated with one-way ANOVA Tukey’s multiple comparison test.


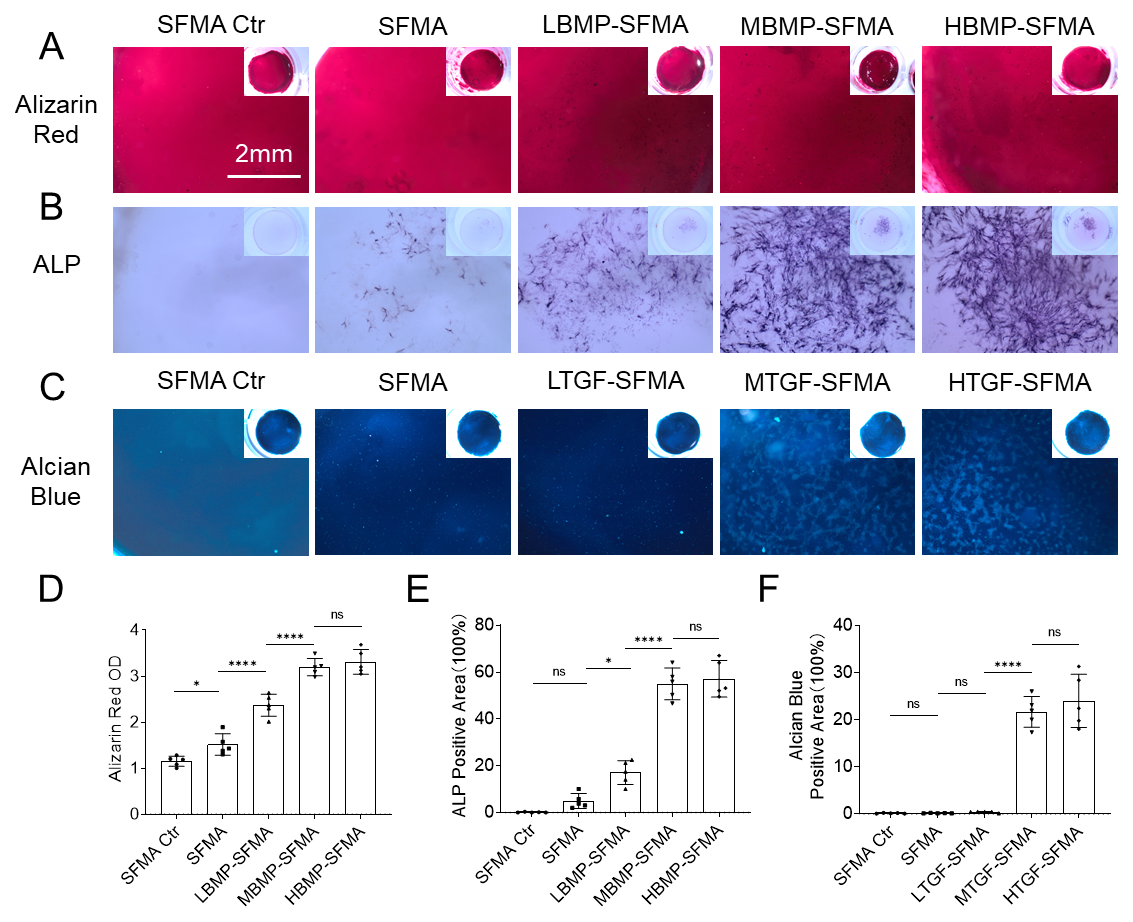


**Figure S5 |The osteogenic and chondrogenic ability of bioactive glues.** Alizarin Red staining (A) and ALP staining(B) of SFMA Ctr, SFMA, LBMP-SFMA, MBMP-SFMA and HBMP-SFMA group. Scale bar represents 2mm.(C) Alcian Blue staining of SFMA Ctr, SFMA, LTGF-SFMA, MTGF-SFMA and HTGF-SFMA group. (D) Quantitative analysis of Alizarin Red staining. (E) Quantitative analysis of ALP staining. (F) Quantitative analysis of Alcian Blue staining. SFMA Ctr (200μl SFMA incubated without rabbit BMSCs), LBMP-SFMA (200μl 20% w/v SFMA with 20μg/ml BMP-2), MBMP-SFMA (200μl 20% w/v SFMA with 100μg/ml BMP-2), HBMP-SFMA (200μl 20% w/v SFMA 500μg/ml BMP-2), LTGF-SFMA (200μl 20% w/v SFMA with 20ng/ml TGF-β3), MTGF-SFMA (200μl 20% w/v SFMA with 100ng/ml TGF-β3), HTGF-SFMA (200μl 20% w/v SFMA with 500ng/ml TGF-β3). Significant differences are presented, exact p-value was calculated with one-way ANOVA Tukey’s multiple comparison test.


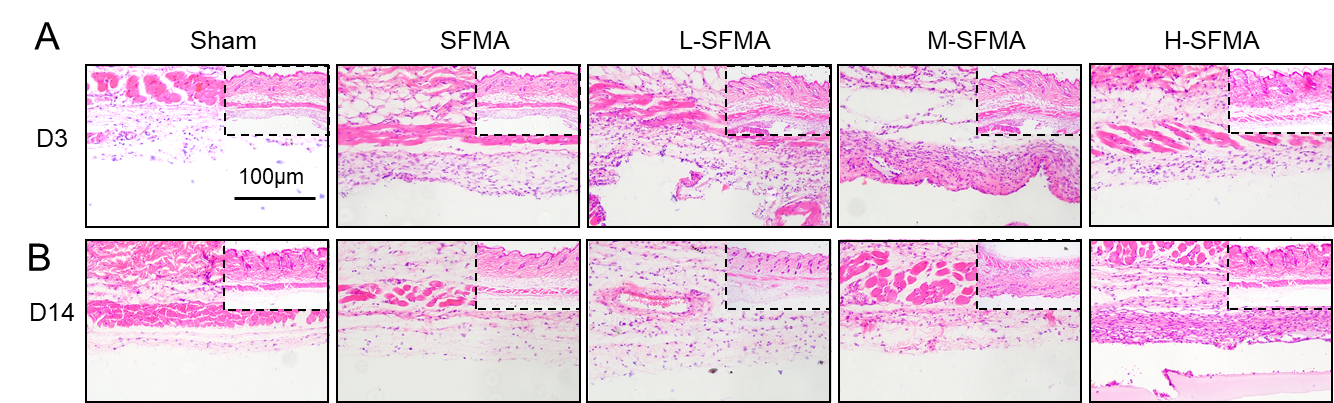


**Figure S6 | The local toxicity of bioactive glues in mice subcutaneous implantation.** 7(A) and 14(B) days post subcutaneous implantation. L-SFMA (200μl 20% w/v SFMA with 20ng/ml TGF-β3 and 20μg/ml BMP-2), M-SFMA (200μl 20% w/v SFMA with 100ng/ml TGF-β3 and 100μg/ml BMP-2), H-SFMA (200μl 20% w/v SFMA with 500ng/ml TGF-β3 and 500μg/ml BMP-2).


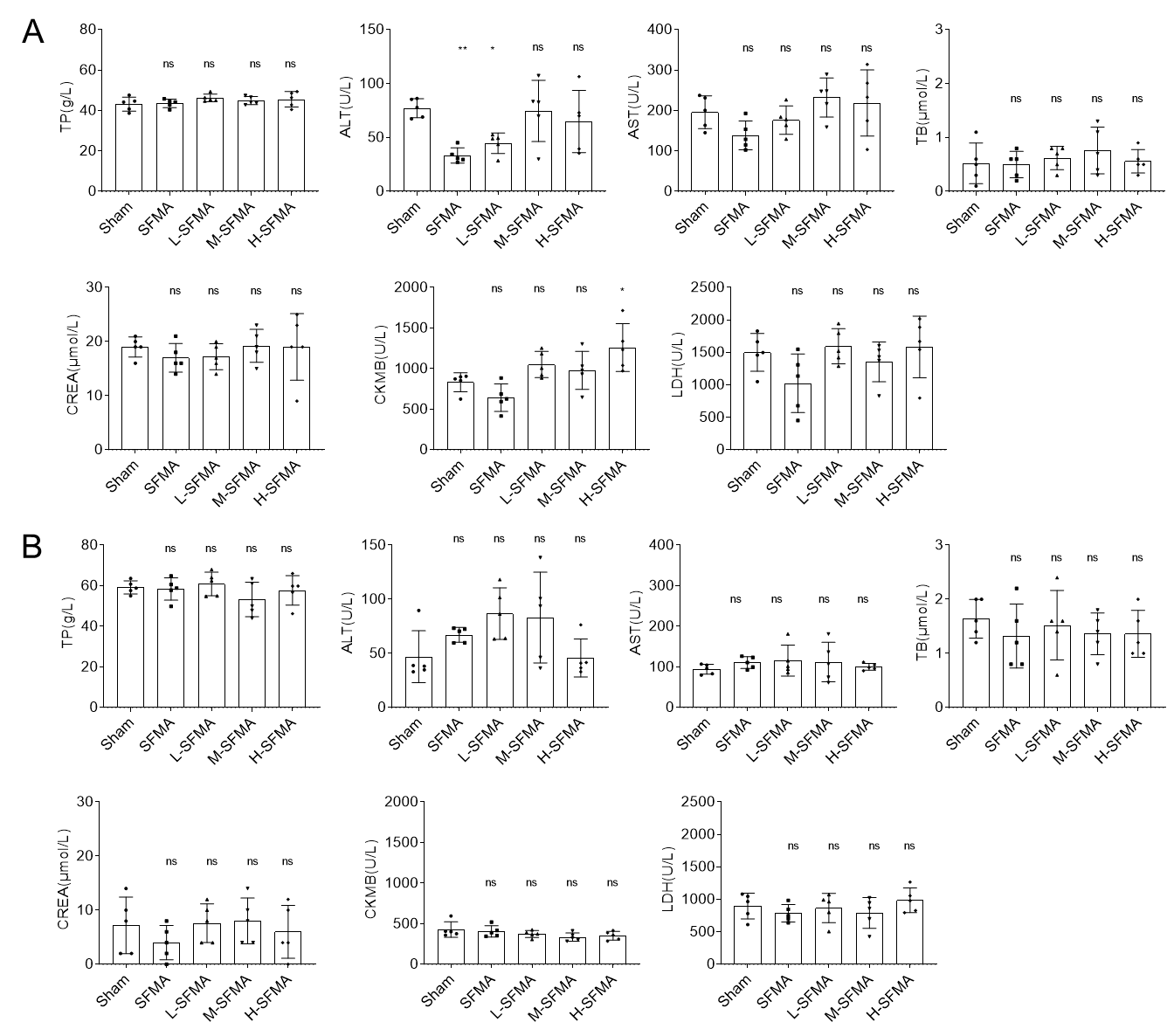


**Figure S7 | Biochemical parameters after bioactive glues implantation in mice.** (A) Biochemical parameters of blood serum collected 3 days after implantation of bioactive glues, indicating that M-SFMA did not induce significant toxicity in a short-term observation. TP, total protein; ALT, Alanine Aminotransferase; AST, Aspartate Aminotransferase; TB, total bilirubin; CREA, creatinine; CKMB, Creatine Kinase MB; LDH, Lactate Dehydrogenase. Sample = 5 for each group. (B) Biochemical parameters of blood serum collected 14 days after implantation of bioactive glues, indicating that M-SFMA did not induce significant toxicity in a long-term observation. Significant differences are presented, exact p-value was calculated with one-way ANOVA Tukey’s multiple comparison test.


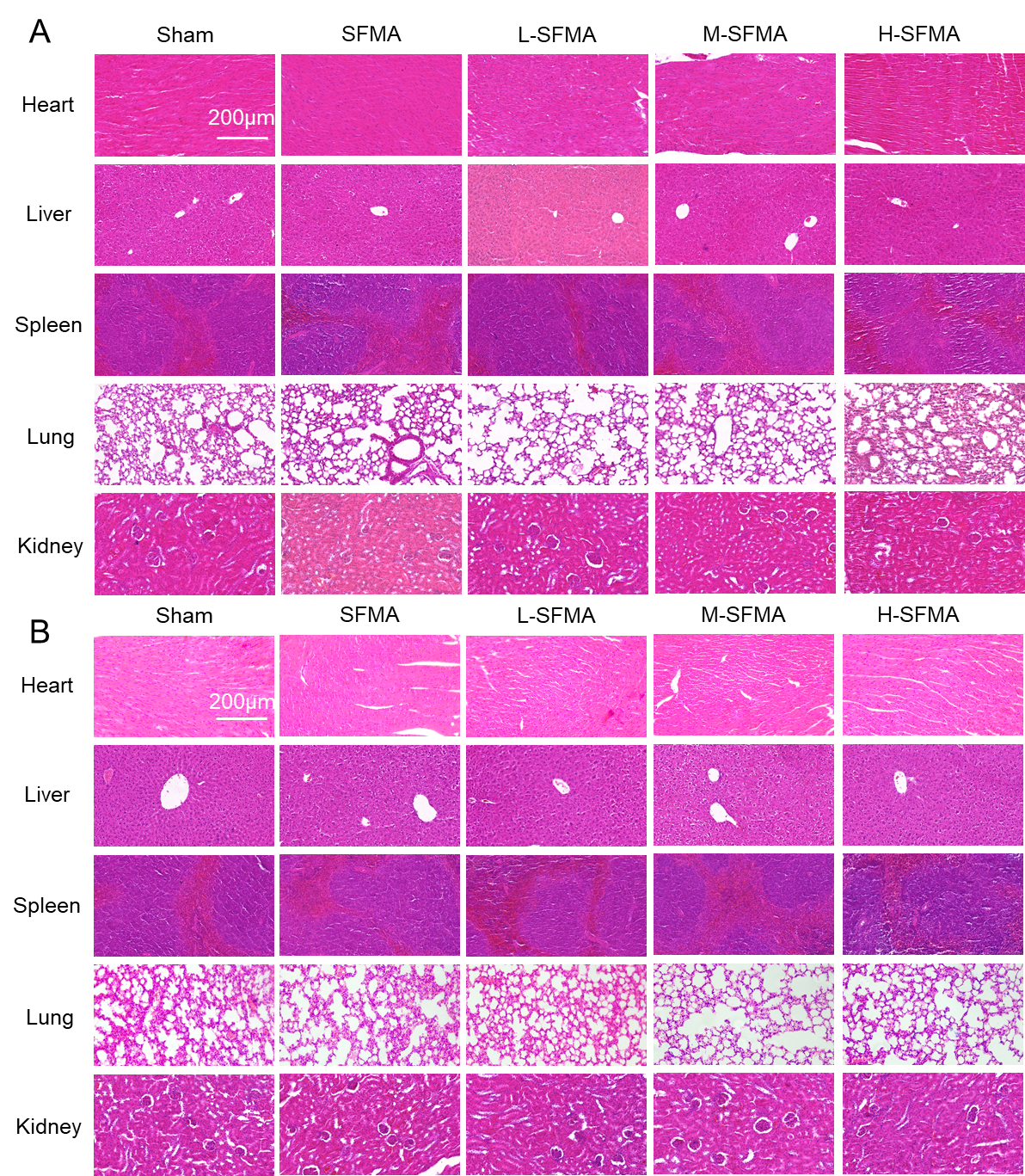


**Figure S8 | Biocompatibility evaluation of bioactive glues in mice hearts, livers, spleens, lungs and kidneys.** H&E staining shows M-SFMA did not significantly induce short-term(A) and long-term(B) inflammation, hemorrhage and necrosis in mice hearts, livers, spleens, lungs and kidneys; scale bar: 200 μm.


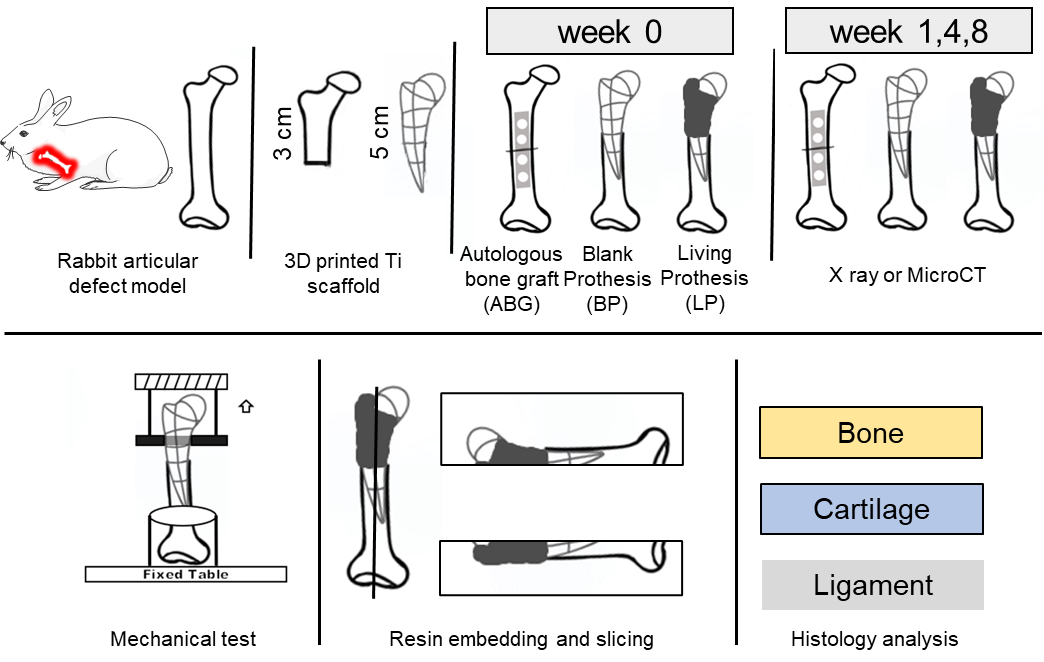


**Figure S9| The procedure of living prothesis evaluation.**


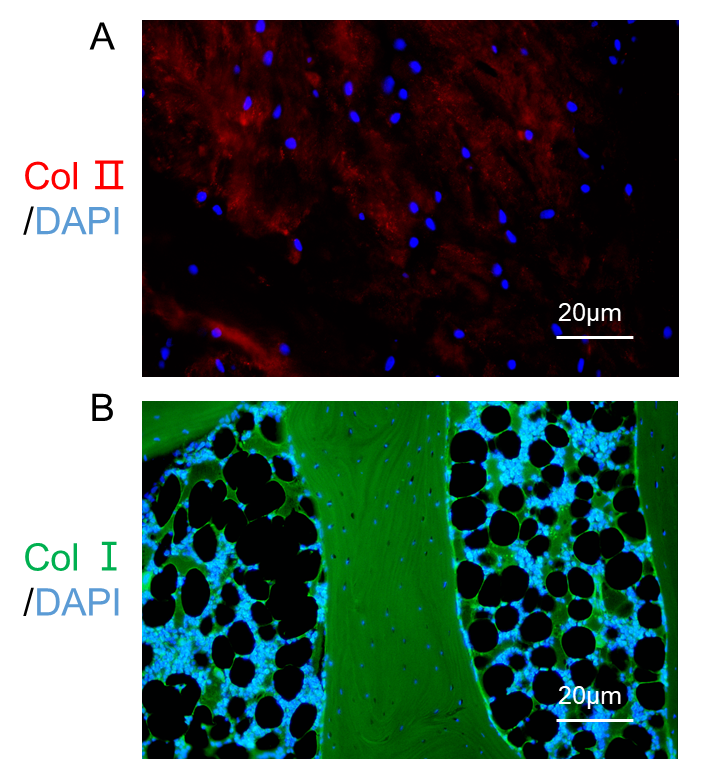


**Figure S10 | The immunofluorescence of regenerated tissue in LP group.** (A) Collagen Ⅱ immunofluorescence of cartilage in LP group. (B) Collagen Ⅰ immunofluorescence of bone in LP group


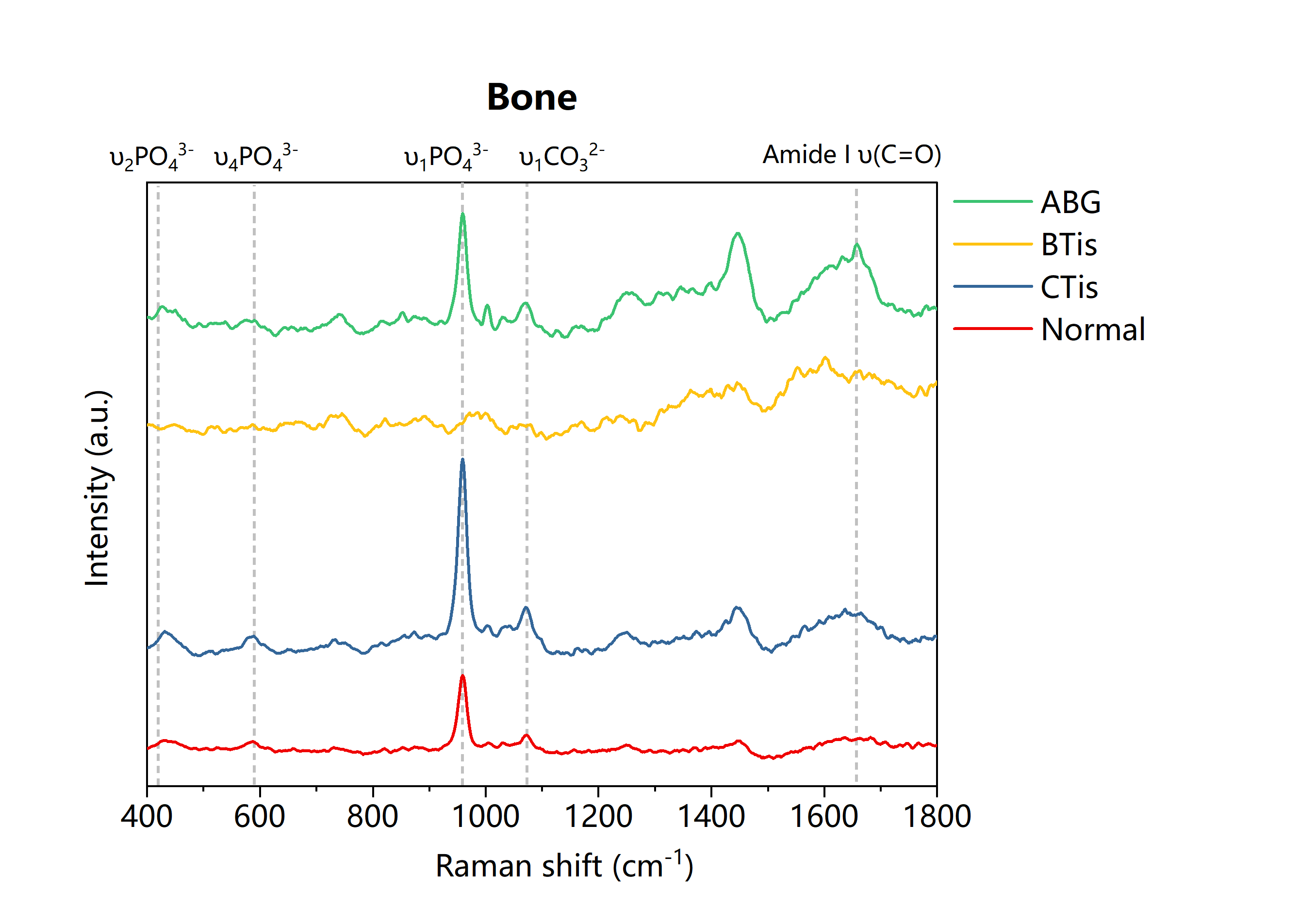


**Figure S11 | Raman microscopy of regenerated cortical bone.**


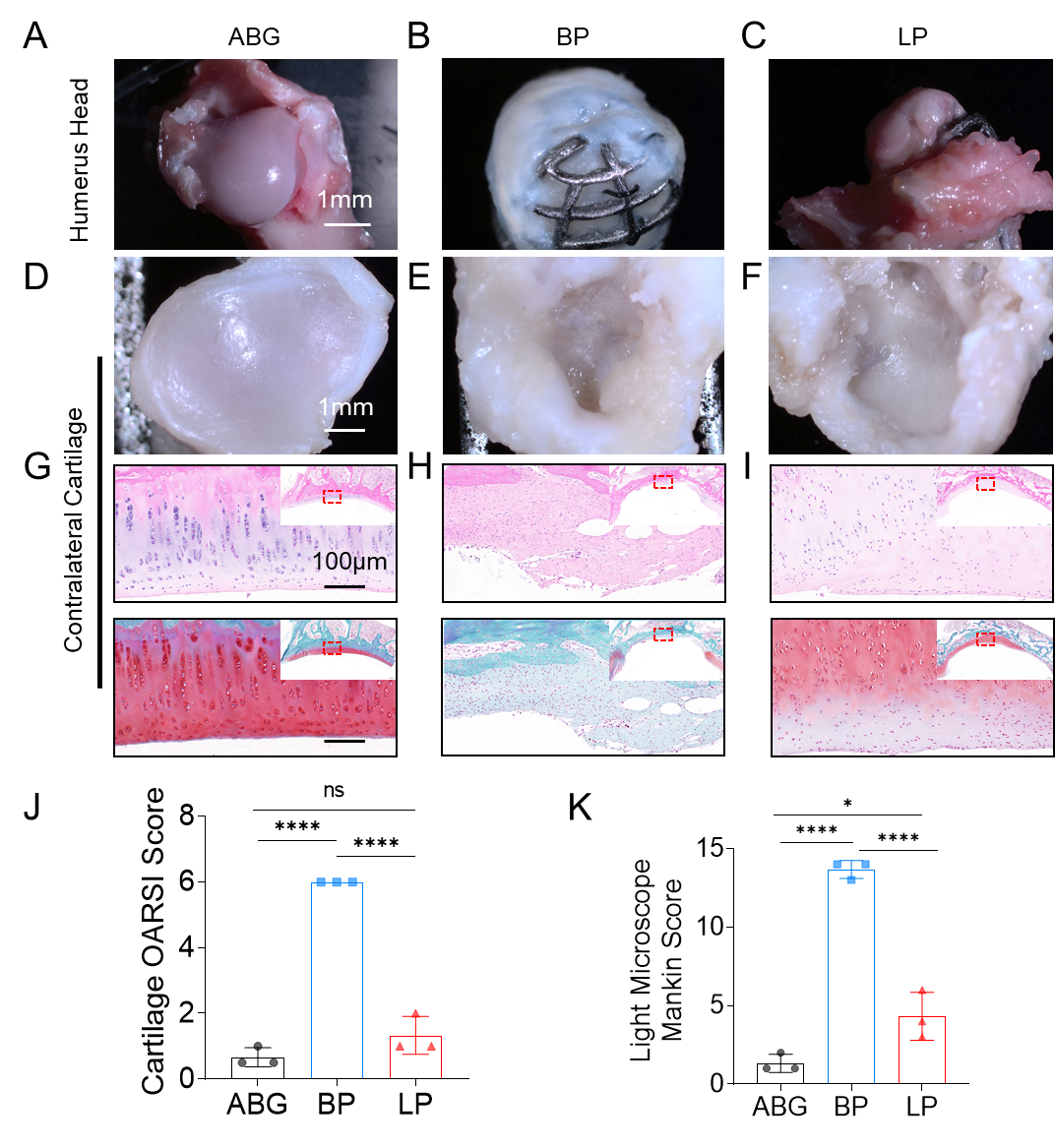


**Figure S12 | Images of humerus head and** **contralateral cartilage.** (A)(B)(C)The gross view of humeral head specimens in vivo after 2 months of total joint replacement. (D)(E)(F)Gross view of contralateral cartilage in vivo after 2 months of total joint replacement. (G)(H)(I) Hematoxylin-Eosin Staining(H&E) and Safarin O/Fast green staining(SO/FG) of contralateral cartilage.(J) Cartilage ICRS scores of contralateral cartilage by histological analysis. (K)Mankin score of contralateral cartilage by light microscope. Significant differences are presented, exact p-value was calculated with one-way ANOVA Tukey’s multiple comparison test.


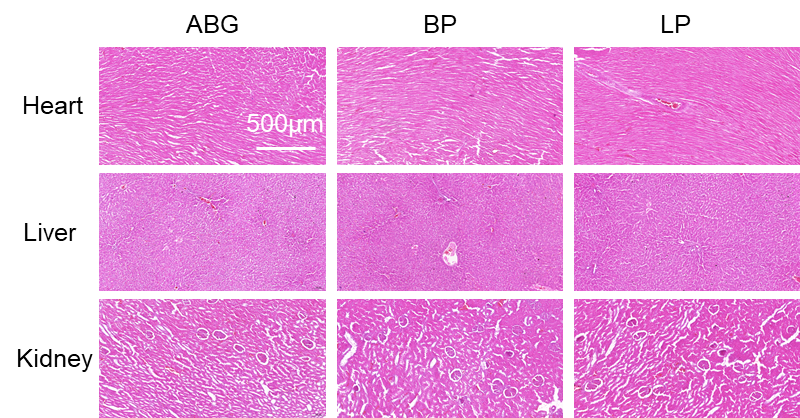


**FigureS13 | HE staining of heart, liver and kidney of different groups.** All groups don’t show obvious tissue inflammation, hemorrhage and necrosis.


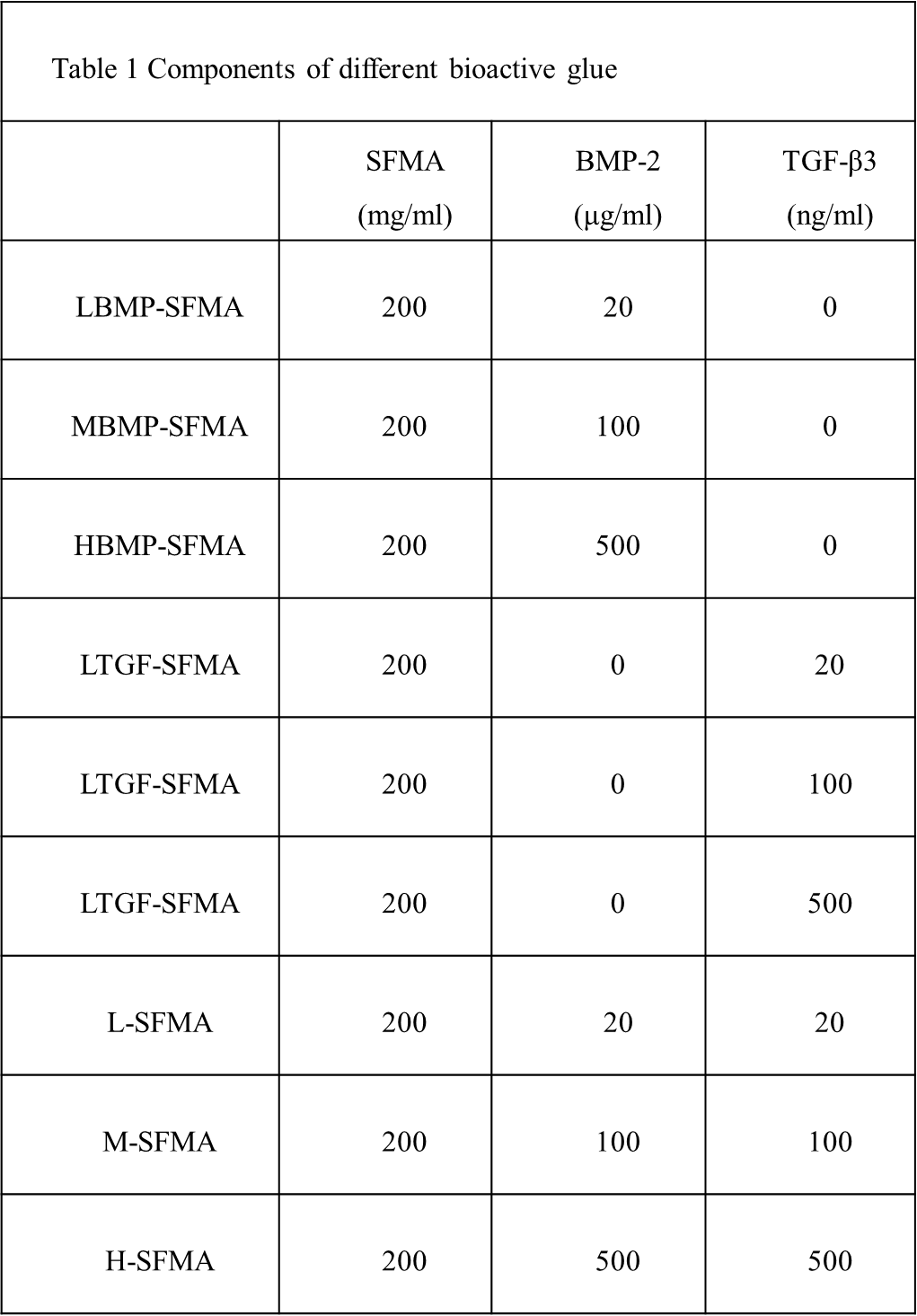


**Table 1 | Components of different bioactive glues.**


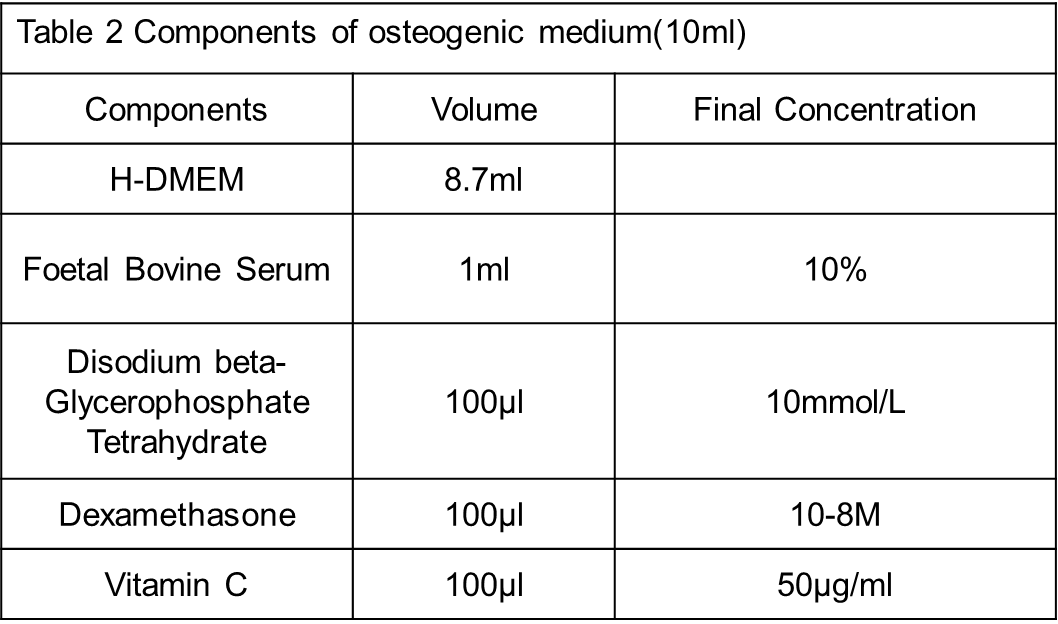


**Table 2 | Components of osteogenic medium.**


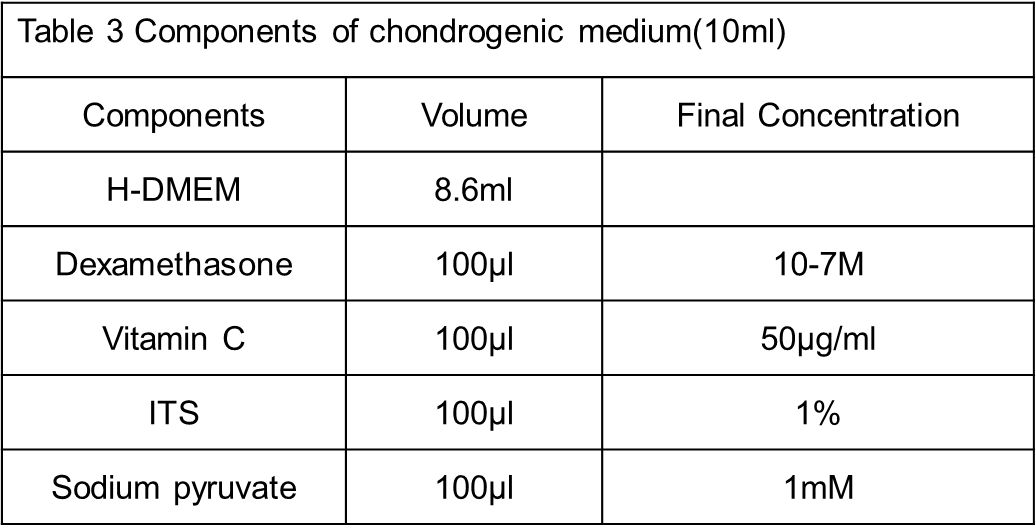


**Table 3 | Components of chondrogenic medium.**
